# Hospitalizations due to rotavirus gastroenteritis in Catalonia, Spain, 2003-2008

**DOI:** 10.1186/1756-0500-4-429

**Published:** 2011-10-20

**Authors:** Alberto L García-Basteiro, Anna Bosch, Elisa Sicuri, José M Bayas, Antoni Trilla, Edward B Hayes

**Affiliations:** 1 Preventive Medicine and Epidemiology Unit. Hospital Clínic. C/Villarroel 170. CP 08036 Barcelona, Spain; 2 Catalan Health Services, Generalitat of Catalonia. Travessera de les Corts, 131-159 Edifici Olímpia, CP 08028 Barcelona, Spain; 3 Barcelona Centre for International Health Research (CRESIB, Hospital Clínic-Universitat de Barcelona), C/Roselló 132, 4/1. CP 08036 Barcelona, Spain

## Abstract

**Background:**

Rotavirus is the most common cause of severe gastroenteritis among young children in Spain and worldwide. We evaluated hospitalizations due to community and hospital-acquired rotavirus gastroenteritis (RVGE) and estimated related costs in children under 5 years old in Catalonia, Spain.

**Results:**

We analyzed hospital discharge data from the Catalan Health Services regarding hospital admissions coded as infectious gastroenteritis in children under 5 for the period 2003-2008. In order to estimate admission incidence, we used population estimates for each study year published by the Statistic Institut of Catalonia (Idescat). The costs associated with hospital admissions due to rotavirus diarrhea were estimated for the same years. A decision tree model was used to estimate the threshold cost of rotavirus vaccine to achieve cost savings from the healthcare system perspective in Catalonia. From 2003 through 2008, 10655 children under 5 years old were admitted with infectious gastroenteritis (IGE). Twenty-two percent of these admissions were coded as RVGE, yielding an estimated average annual incidence of 104 RVGE hospitalizations per 100000 children in Catalonia. Eighty seven percent of admissions for RVGE occurred during December through March. The mean hospital stay was 3.7 days, 0.6 days longer than for other IGE. An additional 892 cases of presumed nosocomial RVGE were detected, yielding an incidence of 2.5 cases per 1000 child admissions. Total rotavirus hospitalization costs due to community acquired RVGE for the years 2003 and 2008 were 431,593 and 809,224 €, respectively. According to the estimated incidence and hospitalization costs, immunization would result in health system cost savings if the cost of the vaccine was 1.93 € or less. At a vaccine cost of 187 € the incremental cost per hospitalization prevented is 195,388 € (CI 95% 159,300; 238,400).

**Conclusions:**

The burden of hospitalizations attributable to rotavirus appeared to be lower in Catalonia than in other regions of Spain and Europe. The relatively low incidence of hospitalization due to rotavirus makes rotavirus vaccination less cost-effective in Catalonia than in other areas with higher rotavirus disease burden.

## Background

Rotavirus gastroenteritis (RVGE) is the most common cause of severe gastroenteritis among children under 5 years of age, causing considerable morbidity and mortality worldwide [1,2]. In Europe, RVGE causes approximately 230 deaths, more than 87,000 hospitalizations and 700,000 outpatient visits annually, consuming substantial health resources [3]. Although costs for treatment of severe cases (i.e. those admitted to hospital) are relevant in Europe [4], rotavirus vaccine is still too expensive to encourage the implementation of a generalized immunization strategy in areas of low incidence [5].

Previous studies regarding the burden of rotavirus disease in Europe and in different autonomous regions within Spain have yielded varying estimates of the number of hospitalizations attributable to rotavirus [6-10]. Few studies in Spain have assessed the burden of nosocomial rotavirus infection, even though it is considered to be one of the principal hospital-acquired infections in young children [9,11-13]. We describe here the characteristics of hospitalizations associated with rotavirus infection in the autonomous region of Catalonia during the years 2003-2008. In addition, we evaluated the cost-effectiveness and threshold vaccine cost for savings with rotavirus vaccination.

## Methods

We analyzed hospital discharge data for Catalonia from the Minimum Basic Data Set for Acute-care Hospitals (MBDS) of the Catalan Health Services. This data set comprises data from all public acute care hospitals and almost 90% of private acute care hospitals.

Diagnoses are coded according to the International Classification of Diseases, 9th revision, Clinical Modification (ICD-9-CM). We analyzed data for all hospitalizations of children less than 5 years of age with a principal diagnosis of infectious gastroenteritis (IGE) to estimate the frequency of community-acquired RVGE that required hospitalization. We defined community-acquired RVGE as all hospitalizations with a primary ICD9 CM code for RVGE (008.61), as well as those with a secondary code for RVGE, when the primary code was an IGE-related code. To evaluate the burden of nosocomial RVGE we selected hospitalizations with primary diagnoses that were not related to IGE but with RVGE coded in any of the secondary diagnoses. We analyzed data regarding the length of hospital stay, age, gender, date of admission, and outcome at discharge. The six-year period evaluated started the 1^st ^of January 2003 and ended the 31^st ^of December 2008.

The following ICD-9-CM codes were selected for IGE: 003.0, 005.0-005.9, 008.0-008.8 and 009.0-009.3. We excluded codes that are most likely to be imported gastroenteritis: cholera (001.0-001.9), typhoid and paratyphoid fever (002.0-002.9), shigellosis (004.0-004.9) and amoebiasis (006.0-006.9), which rarely if ever cause hospitalization in Catalonia. The primary diagnosis was assumed to be the main reason for hospital admission. To estimate the incidence of hospitalizations due to RVGE and other types of IGE, we obtained population estimates for each study year published by the Statistic Institut of Catalonia (Idescat). To estimate the incidence of nosocomial hospitalizations due to RVGE, we obtained the annual number of hospitalizations of children under 5 years of age from the annual reports of MBDS. The average length of stay in hospital was calculated for the different types of IGE and for each of the study years. Statistical analyses and data processing were conducted using STATA v.11.0 and Excel Office 2007.

Total admission costs from the health care perspective were calculated by multiplying the estimated number of cases (community acquired RVGE) of each year by the average rotavirus admission costs. The latter were calculated using the cost of a proxy Diagnosis Related Group (DRG) for this disease (see below), which was obtained from the Spanish Ministry of Health (http://www.msps.es), and weighted using data from the Centers for Medicare and Medicaid Service (CMS-DRG version 22) as in López-de-Andres *et al*[9]. Since there is no specific DRG for rotavirus gastroenteritis, DRG 184 (esophagitis, gastroenteritis, and miscellaneous digestive disorders, age < 18) was used.

Vaccination strategy was compared to "no vaccination" strategy in a decision tree model using the incidence of hospitalizations due to community acquired RVGE and associated costs of one episode estimated in this study. The tree model calculated the expected values associated with each strategy, that is, the costs associated with each strategy weighted by the probability of incurring such costs. In the model the preferred strategy is the one with the lower expected cost. Information on vaccine efficacy was taken from the literature [14] and the current vaccine price was taken from a publication of the Spanish Ministry of Health [15]. Conservatively, vaccine price was assumed to represent all the costs associated with the vaccination program. Most of the input variables were included as probability distributions rather than as point estimates to model uncertainty (Table 1)[16]. Incremental costs per case prevented were calculated (vaccine cost - costs saved due to outcomes averted). The decision tree was built using Tree Age Pro 2008 software (TreeAge Software, Willamstown, MA).

**Table 1:** Input parameters for the decision tree model of the comparison of vaccination vs no vaccination strategies in Catalonia, Spain

Variable	Distribution	Value	Source
Vaccine price* (€)	Point estimate	187	Ruiz, J *et al*. [15]
Vaccine efficacy (%)	Uniform	Low = 81.8, High = 100	Cortese, MM. et al.[14]
Probability of admission due to disease (%)	Triangular	Min = 0.084, Best = 0.104, Max = 0.128	Our estimate
Cost of one episode admitted (€)	Point estimate	2018.02	DRG estimate for the year 2008

* Price for two doses of the vaccine purchased in Spain in March, 2007

### Ethics

This study did not need to pass ethical approval by an ethics committee due to the nature of the data (non experimental). Anonymity was assured as the data provided by the MBDS of the Catalan Health Services did not contain any information that would allow identification of any subject. The guidelines of the Declaration of Helsinki on confidentiality and data privacy were followed.

## Results

### Community Acquired Rotavirus Gastroenteritis

From 2003 to 2008, 10655 children under 5 years of age were hospitalized with IGE as a primary diagnosis in Catalonia (Table 2). These were considered to be community-acquired IGE. The average age of these children was 12.3 months (standard deviation (SD) 13.9 months), and 45% were female.

**Table 2:** Number of infectious gastroenteritis hospitalizations, incidence of hospitalization per 100000 population, and mean hospital stay among children < 5 years old in Catalonia, Spain, 2003-2008

	Gastroenteritis due to Rotavirus				Other Infectious Gastroenteritis				Total Infectious Gastroenteritis			
	
Year	Number	Incidence^1^	Mean stay (SD)^2^		Number	Incidence^1 ^	Mean Stay(SD)^2^		Number	**Incidence**^1^	Mean Stay(SD)^2^	
2003	287	83.6	3.8	(2.4)	1420	413.7	3.4	(2.5)	1707	497.3	3.5	(2.5)
2004	331	91.8	3.9	(2.3)	1489	412.9	3.3	(3.0)	1820	504.7	3.9	(2.9)
2005	481	127.6	3.6	(1.9)	1332	353.5	3.0	(2.4)	1813	481.1	3.2	(2.3)
2006	472	120.3	3.9	(2.0)	1472	375.1	2.9	(2.4)	1944	495.4	3.2	(2.3)
2007	401	98.5	3.7	(3.0)	1427	350.5	2.9	(2.2)	1828	449.0	3.1	(2.4)
2008	401	99.2	3.5	(2.0)	1142	282.5	2.9	(2.7)	1543	381.7	3.1	(2.6)

Period 2003-2008	2373	103.9^3^	3.7	(2.3)	8282	362.5^3^	3.1	(2.5)	10655	466.4^3^	3.2	(2.5)

^1^Incidence per 100000 population

^2^Mean hospital stay in days (standard deviation [SD] in parentheses)

^3 ^Average annual incidence

A total of 2373 hospital admissions had RVGE as either the primary diagnostic code or had RVGE as a secondary code with an IGE-related primary code, and were thus considered community-acquired cases of RVGE. These hospitalizations with RVGE codes represented 22% of all IGE hospitalizations. Ninety-eight percent of these hospitalizations had RVGE coded as the primary diagnosis. On average there were 396 community-acquired RVGE hospitalizations per year, varying from 287 in 2003 to 481 in 2005. Using the average population under 5 years of age during the study period 2003-2008 (380,751 residents) we estimate that the average annual incidence of RVGE hospitalization was 104 admissions per 100,000 children < 5 years, ranging from 84 to 128 in 2003 and 2005 respectively. There were no deaths recorded associated with community-acquired RVGE hospitalizations. Assuming that there are an average of 8 (range 5 to 10) outpatient visits per hospital admission as Parashar and colleagues have suggested [2], we estimate that rotavirus infection causes 3,168 (range 1,980 to 3,960) outpatient visits annually in Catalonia.

**Figure 1:**
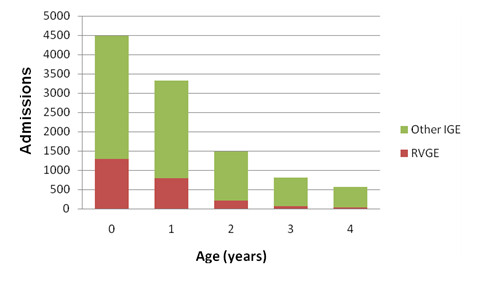
**Distribution by age of hospital admissions in Catalonia, Spain, due to rotavirus gastroenteritis (RVGE) and other infectious gastroenteritis (IGE) among children < 5 years old, 2003-2008**.

The mean age of children hospitalized for RVGE was 7.6 months (SD 10.1 months), and 42% were female. Most (54%) RVGE hospitalizations were of children under 1 year of age and 88% were of children younger than 2 years. The proportion of IGE attributed to rotavirus decreased with age (Figure 1).

**Figure 2:**
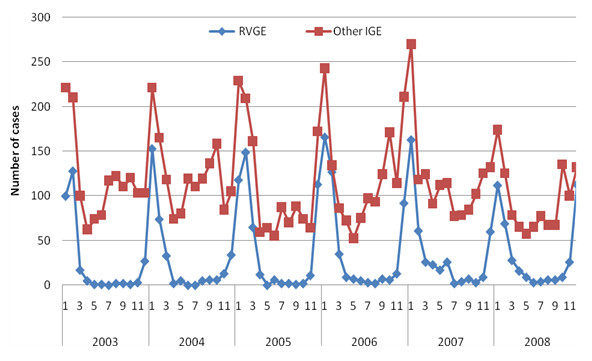
**Hospitalizations due to rotavirus gastroenteritis (RVGE) and other infectious gastroenteritis (IGE) in children under 5 years of age in Catalonia, Spain, by year (2003-2008)**.

The seasonality of RVGE admissions and other IGE admissions is shown in Figure 2. Eighty-seven percent of RVGE admissions occurred in the months of December through March, whereas only 46% of admissions for other IGE occurred during the same months.

The average hospital stay for all children with community-acquired IGE was 3.2 days (SD 2.5 days) ranging from 1 to 65 days with a median of 3.0 days (Table 2). Children with community-acquired RVGE had a slightly longer average stay than those with other IGE: 3.7 days (SD 2.3) vs 3.1 days (SD 2.5).

### Nosocomial Rotavirus Gastroenteritis

During the period 2003-2008, 892 hospitalizations of children less than 5 years old had secondary diagnoses of RVGE with primary diagnoses that were not IGE-related (Table 3). Hospital-acquired RVGE represented 27% (892/3,265) of all diagnoses of rotavirus infection. Using the total number of hospitalizations of children under 5 years of age during the study period (355,339 hospitalizations) we estimate that the average annual incidence of presumed nosocomial RVGE was 2.5 cases per 1,000 hospitalizations < 5 years, ranging from 2.0 to 2.8 in 2007 and 2004 respectively. Forty-five percent of the children with presumed nosocomial RVGE were females; 66% were less than 1 year of age and 90% were less than 2 years old. The mean age was 5.8 months (SD 9.4), lower than for community-acquired RVGE.

**Table 3:** Number of suspected hospital-acquired rotavirus gastroenteritis, annual incidence, and mean hospital stay in children < 5 years old in Catalonia, Spain, 2003-2008

Year	Number	Incidence^1^	Mean Stay (SD)^2 ^
2003	149	2.6	12.1(18.8)
2004	165	2.8	8.1(11.8)
2005	148	2.5	10.6(14.2)
2006	154	2.6	10.1(15.1)
2007	124	2.0	10.3(11.1)
2008	152	2.5	7.2(6.8)

Period 2003-2008	892	2.5	9.7(13.6)

^1^Incidence per 1000 hospital admissions.

^2^Mean hospital stay in days (standard deviation [SD] in parentheses)

The mean hospital stay for children with presumed nosocomial RVGE was 9.7 (SD 13.6) days and the median was 6 days. Children under one year of age and those 4 years old had longer hospital stays than the rest: mean stay 10.6 (SD15.1) and 14.0 (SD 12.8) days respectively vs. 7.6 (SD 8.0), 8.3(SD 13.5) and 8.0 days (SD 5.6) for 1, 2 and 3 year-old children respectively. There was one death associated with presumed nosocomial RVGE in 2006.

### Costs and decision tree model

**Figure 3:**
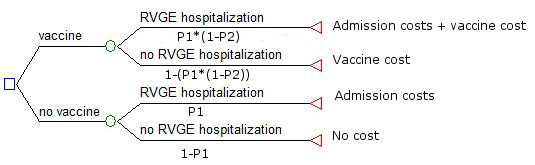
**Decision tree model of the comparison of vaccination vs no vaccination in Catalonia, Spain**. *P1 = incidence of RVGE hospitalization; P2 = Vaccine efficacy.

The structure of the decision tree model is depicted in Figure 3. Costs for 1 episode (admission) range from 431,593€ in the year 2003 to 861,833€ in the year 2006 (table 4). At the current rotavirus vaccine price (187 €-two doses) the least expensive strategy is "no vaccine", with an estimated expected cost of 2.13 € per person compared to an estimated expected cost of 187.19 € per person associated with vaccination. The two strategies become equivalent with an expected cost of 2.1 € per person, when the vaccine cost is reduced to 1.93 €. The incremental cost per case prevented associated with the vaccination strategy was estimated to be of 195,388 € (CI 95% 159,300; 238,400).

**Table 4:** Hospitalizations costs of community acquired rotavirus gastroenteritis in our study period

Year	Estimated cost per episode*	Number of rotavirus hospitalizations	Total cost (euros)
2003	1,503.81	287	431,593.47
2004	1,639.64	331	542,720.18
2005	1,746.36	481	839,999.16
2006	1,825.92	472	861,832.54
2007	1,935.55	401	776,155.15

2008	2,018.02	401	809,224.42

* Cost estimated using DRG 184 (DRG: Diagnosis Related Groups)

## Discussion

The lack of a specific surveillance system for RVGE makes it difficult to directly estimate the burden of this disease in Spain. The percentage of IGE hospital admissions due to rotavirus in Spain ranged from 31% to 45% in previous studies with similar methodology to ours [8-10]. We found that 22% of the IGE hospital admissions in children under 5 in Catalonia, from 2003 through 2008, were due to rotavirus infection.

The incidence of admissions for rotavirus during our study period, 104 per 100,000 children < 5 years, is also lower than the most recent estimate for the total of Spain of 135 cases per 100000, and lower than recent estimates for other European countries [3,7,9,17-19]. Our estimate is also lower than that from a previous study conducted in Catalonia (study period 1999-2000) of 125 per 100000 [20]. The lower estimates in Catalonia compared to the rest of Spain could reflect actual geographic variability in incidence of rotavirus infection or disease, or could be due to differences in the sensitivity of the hospital discharge databases for detecting RVGE. Similarly, the apparent decreased incidence we found compared to the earlier estimate for Catalonia by Gil et al. might reflect a true decreased incidence over the years since 2005 or could be due to changes in detection sensitivity. However, the coverage of MBDS for public hospitals improved from 95% to 100% over the time between the two estimates [20].

The seasonality of RVGE has already been widely described by many studies in Europe and America, and our data also shows increased incidence during winter months. During the years we evaluated, there was a progressive increase of incidence through 2005, which is consistent with results of previous evaluations in Spain [9]. At least some of the increase could have been due to enhanced clinical awareness. The slight decrease in hospital admissions in 2007 and 2008 might be attributed to the introduction of the rotavirus vaccines in the Spanish market during 2006 and 2007. However, based on numbers of vaccine doses sold to pharmacies in Catalonia by pharmaceutical companies, we estimated vaccine coverage among children less than 1 year old at only 20% in 2008. The coverage among children up to 5 years of age would be much lower, thus limiting the impact of vaccination on hospitalization to date.

We found an average of 0.6 day longer mean hospital stay for hospitalizations due to RVGE compared with hospitalizations for other causes of IGE. Similar results were described in other studies using Vesikari's score to account for duration and severity of diarrhea [21,22]. Thus we estimate that if there had been vaccination coverage similar to that for measles, mumps and rubella vaccine (approximately 96%)[23], and a 90% efficacy of a rotavirus vaccine [24,25], over 7500 days of hospitalization attributable to rotavirus could have been prevented over the 6 year study period. Achieving high vaccine coverage would likely require inclusion of rotavirus vaccine in the national immunization program.

The average annual incidence of presumed nosocomial RVGE in our study period was 2.5 per 1000 hospitalizations in children < 5 years of age, which is lower than the incidence of 4.5 per 1000 admissions reported in another recent study for the whole of Spain [26]. However, in that study, the incidence for Catalonia of 2.0 cases per 1,000 children under 5 is quite consistent with our estimate and thus the incidence of nosocomial RVGE in Catalonia appears to be lower than in the rest of Spain. Our finding that children under 12 months old appear to be at higher risk of acquiring nosocomial RVGE than older children, is consistent with previous studies [26,27].

In the economic evaluation of RVGE, the strategy "no vaccine" was substantially less expensive than the vaccination strategy at the current vaccine price of 187 €. At a vaccine cost of 1.93 € the two strategies had the same expected cost. A study from the United States estimated the median cost-effectiveness ratio of vaccination program per serious case prevented at 3024 US$ [28], substantially lower than our estimate for Catalonia. Thus, rotavirus vaccination in Catalonia does not appear to be as cost-effective as in the United States. The cost-effectiveness ratio estimated in our study is consistently higher due to the lower probability of hospitalization, higher vaccine price and lower costs of hospitalization.

There are limitations to using the MBDS to estimate RVGE incidence and severity. First, the dataset covers all public hospitals in our region but it may miss cases of RVGE that are not laboratory confirmed. Second, a recent study in Italy indicated that 27% of RV positive cases admitted to the hospital were not coded as RVGE in the hospital discharge database, and two studies in Spain found that 52% (Foster J. et al) and 47% (Gutierrez-Gimeno et al) of children hospitalized for gastroenteritis in Spain and prospectively tested for rotavirus infection were positive [21,29,30]. Similar coding and diagnostic error in our dataset would result in underestimation of the true number of RVGE cases. However, the MBDS system in Catalonia uses an internal and external validation system that monitors and updates the hospital discharge data before processing and storage, thus capturing diagnoses of cases that were discharged before the results of in-hospital microbiological tests. Third, there might be cases of nosocomial RVGE that are not detected if the symptoms start after discharge and do not provoke re-hospitalization. On the other hand, our definition for presumed nosocomial RVGE might lack specificity if the primary diagnostic code does not reflect the actual cause of admission and this would lead to labeling some cases of community-acquired RVGE as presumed nosocomial RVGE, resulting in an overestimate of nosocomial RVGE. Fourth, some hospitals might not run diagnostic tests for RVGE as frequently as others, yielding an underestimation of RVGE cases. Fifth, our economic evaluation only concerns community acquired RVGE and is calculated from the health system perspective, and therefore does not consider indirect costs associated with rotavirus admissions (such us travelling expenses and family members' days off from work). This would underestimate the total societal costs of each admission. However, such indirect costs are likely to be small compared to the direct costs of hospitalization [30]. Lastly, we have not taken into account the unknown rate of readmission for RVGE in our study population. Since the data from MBDS is anonymous, we could not distinguish if the same child was hospitalized more than once. Counting readmissions in our analysis could result in overestimation of the true incidence of both community acquired and nosocomial RVGE. However, since primary rotavirus infection generally protects against subsequent severe rotavirus illness, we believe that our estimates of the incidence of hospital-acquired and nosocomial RVGE are not substantially biased by readmissions.

## Conclusions

In conclusion, rotavirus is an important cause of infectious gastroenteritis associated with hospitalization among children under 5 years old in Catalonia, and especially among children under 2 years of age. RVGE occurs mainly in winter months and causes longer hospital stays than gastroenteritis caused by other infectious agents. However, the incidence of community acquired and nosocomial RVGE in Catalonia appears to be lower than in other regions of Spain and other European countries. The cost of rotavirus vaccination would need to be substantially lower than the current cost in order to make rotavirus vaccination as cost-effective in Catalonia as in many other areas where universal vaccination has been implemented.

## Competing interests

JMB has investigated vaccines of GlaxoSmithKline and Sanofi Pasteur MSD (papilloma virus vaccine and others vaccines). ALGB, AB, ES and EBH have no conflicts of interest to declare.

## Authors' contributions

ALGB performed data analysis and interpretation of the data and drafted the manuscript. AB collected the data and revised the final manuscript. ES participated in the analysis of the paper and drafted the paper. JMB and AT helped in the interpretation of the results and revised the manuscript, provided direction and helped revise the final manuscript. EBH supervised and conceived of the study and drafted the manuscript. All authors read and approved the final manuscript.
